# Pulmonale Rundherde und Pneumonie: Ein diagnostischer Leitfaden

**DOI:** 10.1007/s00117-021-00953-w

**Published:** 2022-01-12

**Authors:** Thomas Frauenfelder, Anna Landsmann

**Affiliations:** Institut für Diagnostische und Interventionelle Radiologie, Universitätsspital Zürich, Rämistr. 100, 8091 Zürich, Schweiz

**Keywords:** Lunge, Infektion, Computertomographie, Röntgenbildgebung, Thorax, Lung, Infections, Computed tomography, Thoracic radiography, Thorax

## Abstract

**Hintergrund:**

Das konventionelle Röntgenbild zählt zu den am häufigsten durchgeführten radiologischen Untersuchungen. Seine Interpretation gehört zu den Grundkenntnissen jedes Radiologen.

**Fragestellung:**

Ziel dieses Artikels ist es, häufige Zeichen und Muster der Pneumonie sowie Merkmale von Pseudoläsionen im konventionellen Röntgenbild zu erkennen und einen diagnostischen Leitfaden für junge Radiologen zu schaffen.

**Methoden:**

Analyse aktueller Studien und Daten sowie eine Übersicht der häufigsten Zeichen und Muster im konventionellen Röntgenbild.

**Ergebnisse:**

Die Kenntnis über häufige Zeichen und Muster im Röntgenbild bietet eine Hilfestellung in der Diagnostik und kann hinweisend für die Ursache einer Infektion sein. Häufig sind diese Zeichen jedoch unspezifisch und sollten daher immer in klinische Korrelation gesetzt werden. In der Detektion und Beurteilung von pulmonalen Rundherden gewinnt die Computertomographie (CT) durch ihre deutlich höhere Sensitivität in der Primärdiagnostik immer mehr an Bedeutung.

**Schlussfolgerung:**

Das konventionelle Röntgenbild bildet weiterhin eine führende Rolle in der Primärdiagnostik; der Radiologe sollte jedoch die Limitationen des konventionellen Bildes kennen.

## Abstract (en)

**Background:**

Chest X‑ray is one of the most frequent examinations in radiology and its interpretation is considered part of the basic knowledge of every radiologist.

**Objectives:**

The purpose of this article is to recognize common signs and patterns of pneumonias and pseudonodules in chest X‑rays and to provide a diagnostic guideline for young radiologists.

**Materials and methods:**

Recent studies and data are analyzed and an overview of the most common signs and patterns in chest X‑ray is provided.

**Results:**

Knowledge about common signs and patterns in chest X‑ray is helpful in the diagnosis of pneumonias and can be indicative for the cause of an infection. However, those signs are often unspecific and should, therefore, be set in clinical content. Computed tomography is becoming increasingly important in the primary diagnosis of pulmonary lesions because of its much higher sensitivity.

**Conclusion:**

Chest X‑ray is still the first-line modality in the diagnosis of pneumonia and pulmonary nodules; however, radiologists should be aware of its limitations.

Seit Entdeckung der Röntgenstrahlung im Jahr 1895 zählt das konventionelle Röntgenbild zu den häufigsten durchgeführten radiologischen Untersuchungen. Günstig und schnell verfügbar, verschafft es dem Radiologen eine schnelle Übersicht und ist daher unverzichtbar. Mit etwa 9 % der Untersuchungen, steht die Röntgen-Thorax-Aufnahme an Platz 3 der jährlich durchgeführten Röntgenuntersuchungen, trägt jedoch durch ihre vergleichbar geringe Strahlendosis nur zu 1 % der jährlichen Strahlenbelastung in der Bevölkerung bei [1].

Nichtsdestotrotz ist das Röntgenbild in seiner diagnostischen Sensitivität deutlich eingeschränkt und variiert, je nach Quelle, sehr stark. Während für die Pneumonie eine Sensitivität von 32–78 % berichtet wird, liegt die Spezifität bei 60–94 % [2, 3]. Beim Nachweis pulmonaler Läsionen liegt die angegebene Sensitivität mit 18–46 % wesentlich darunter und zeigt eine deutliche Abhängigkeit von der Erfahrung des Untersuchers, weshalb sie für Screening-Untersuchungen ungeeignet ist [4, 5].

Seine Interpretation, aber auch das Wissen über die Limitationen des Röntgenbildes gehören daher zum Standardwissen jedes Radiologen. Ziel dieses Artikels ist es, sowohl charakteristische Zeichen und Muster für Pneumonien und Pseudoläsionen im Röntgenbild zu erkennen als auch einen diagnostischen Leitfaden für junge Radiologen zu schaffen.

## Pneumonien – die häufigsten Zeichen

Die Pneumonie ist eine der häufigsten erworbenen Infektionen im ambulanten Umfeld und stellt die dritthäufigste Ursache für eine Hospitalisierung dar. Obwohl die Behandlungsmöglichkeiten sich in den letzten Jahrzehnten deutlich verbessert haben, gehört sie mit 14 % Letalität unter den hospitalisierten Patienten zu einer der häufigsten infektionsassoziierten Todesursachen [6, 7]. Laut statistischem Bundesamt starben im Jahr 2019 deutschlandweit rund 18.500 Menschen aufgrund einer Pneumonie [8].

Die Diagnose der Pneumonie ist prinzipiell eine klinische; die konventionelle Aufnahme dient insbesondere dazu, das Ausmaß der Erkrankung und den Therapieerfolg zu eruieren. Das Erscheinungsbild einer Pneumonie kann jedoch stark variieren. Insbesondere in den Frühphasen können Veränderungen im Röntgenbild fehlen. Studien haben gezeigt, dass 15 % der Pneumonien von Radiologen übersehen werden; Heussel et al. berichten sogar, dass 50 % der Patienten mit einer in der Computertomographie (CT) bestätigten Pneumonie keine charakteristischen Zeichen im Röntgenbild aufweisen [9]. Insbesondere die Art des Verteilungsmusters kann, in sehr beschränktem Ausmaß, jedoch hinweisend auf die Art des Erregers sein und somit dem Kliniker eine Hilfestellung für die weitere Therapie bieten [10].

**Figure 1:**
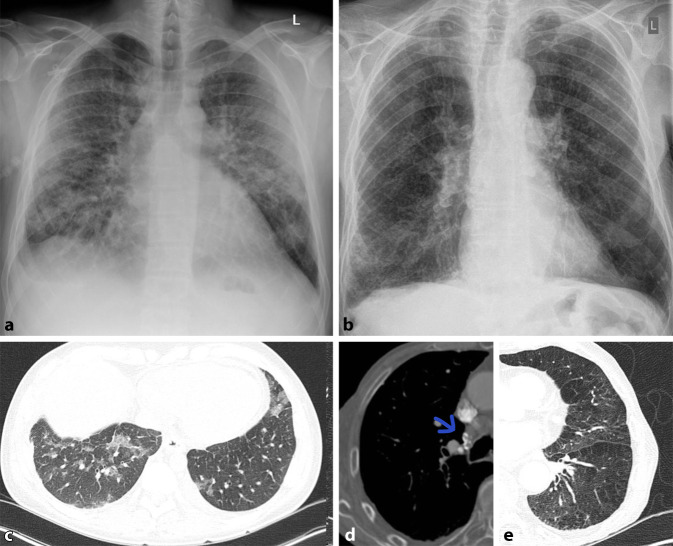

Allgemein unterscheidet man bei Pneumonien interstitielle von azinären Transparenzminderungen. Interstitielle Pneumonien zeigen in ihrem Erregerspektrum eine große Überlappung mit atypischen Pneumonien, weshalb der Begriff häufig synonym verwendet wird. Interstitielle Veränderungen zeigen ein retikulonoduläres (retikulär = netzartig, nodulär = knotig) Muster (Abb. 1). Differenzialdiagnostisch ist hier vor allem die Abgrenzung zu interstitiellen Lungenerkrankungen von Bedeutung. Die Sensitivität des konventionellen Bildes wird bei interstitiellen Pneumonien mit nur 40 % berichtet. Bei entsprechendem Risikoprofil, wie Immunsuppression oder Berufsexposition wird daher immer häufiger eine CT zur Primärdiagnostik eingesetzt [11].

Häufige Erreger der interstitiellen Pneumonie sind Viren oder Pilze. Sie grenzen sich in ihrem Erscheinungsbild von bakteriellen Erregern ab und erzeugen milchglasartige Veränderungen, welche zumeist bilateral auftreten. Solche Ground-glass-Opazitäten werden aufgrund ihrer geringeren Dichte im Röntgenbild jedoch häufig übersehen.

**Figure 2:**
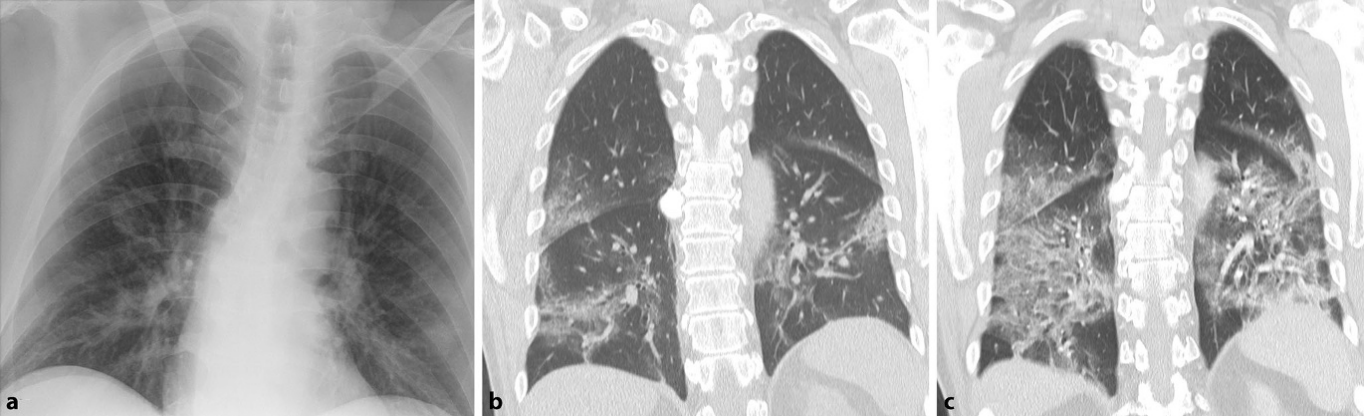

Seit Beginn der COVID-19-Pandemie wächst die Bedeutung der Ground-glass-Opazitäten stetig und eine frühe Diagnose ist hier besonders wichtig. Die typischen peripher betonten milchglasartigen Veränderungen sind insbesondere im Anfangsstadium der Erkrankung im konventionellen Bild häufig nur schwer erkennbar; doch eine frühe Diagnose kann für den Patienten entscheidend sein. Aufgrund geringer Übereinstimmung zwischen schwerer Symptomatik und fehlenden Veränderungen in der konventionellen Bildgebung, gilt es, gleichzeitig Differenzialdiagnosen wie eine Lungenembolie schnell und effizient auszuschließen; auch hier ist eine CT-Untersuchung in der Primärdiagnostik unverzichtbar ([12]; Abb. 2).

**Figure 3:**
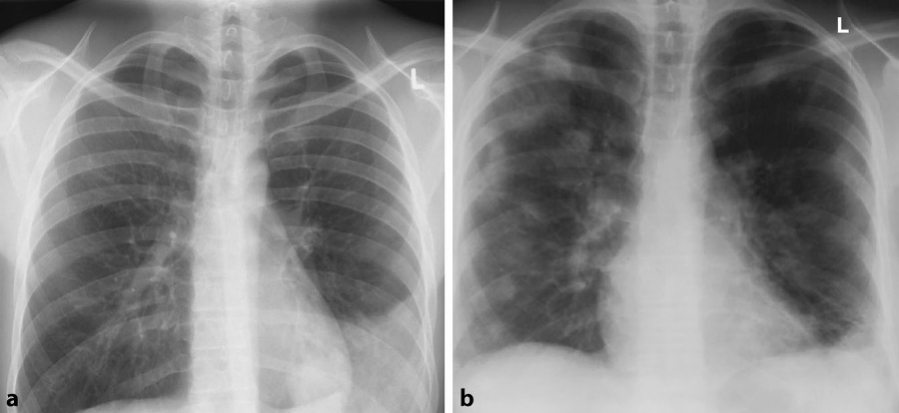

Azinäre oder alveoläre Verschattungen können sich auf einen Lungenlappen (Lobärpneumonie) oder einzelne Lungensegmente (Bronchopneumonie) beschränken (Abb. 3). Nicht nur klinisch, sondern auch in ihren bildmorphologischen Zeichen lassen sich diese Pneumonien von der interstitiellen Pneumonie unterscheiden (Tab. 1).

**Table 1:** 

	Charakteristika	Erreger
Lobärpneumonie	Homogene Konsolidierungen einzelner Lungensegmente oder des ganzen Lappens, begrenzt durch Lappenspalten Positives Aerobronchogramm Beginnt peripher, setzt sich nach zentral fort Häufig basale Lungenabschnitte befallen Häufig Volumenzunahme des betroffenen Lungenlappens „Bulging fissure sign“ = konvexe Konfiguration der Lappenspalten bei Klebsielle-Pneumonie	*Streptococcus pneumoniae* *Klebsielle pneumoniae* *Legionella pneumophila* *Haemophilus influenza*
Bronchopneumonie = lobuläre Pneumonie	Unscharf begrenzte Noduli und fleckförmige Konsolidierungen oder Milchglastrübungen entlang der Lungensegmente Häufig mehrere Lungenlappen befallen Kein Aerobronchogramm Häufig Atelektasen durch Verlegung der Atemwege	*Staphylococcus aureus* *Streptococcus pneumoniae* *E. coli, Haemophilus influenza* *Pseudomonas aeroginosa* Viren, Mykoplasmen, Pilze
Interstitielle Pneumonie	Häufig bilateraler, diffuser Befall Mischbild aus Milchglastrübungen, Konsolidierungen und retikulonodulären Veränderungen	Viren, Mykoplasmen, Pilze Chlamydien, Rickettsien

*Konsolidierungen* sind alveoläre Füllungsprozesse, bei denen Luft durch Flüssigkeiten wie Mukus ersetzt wird. Die höhere Dichte führt zu einer verstärkten Abschwächung der Röntgenstrahlung und dadurch zu einer Verschattung. Dabei können Grenzen von Gefäßen und Bronchialwänden maskiert werden. Konsolidierungen zählen zu den häufigsten Zeichen der Pneumonie. Während sich diese Verschattungen bei einer Lobärpneumonie zunächst auf einen Lappen beschränken, bildet sich bei der Lobärpneumonie häufig das Muster *fleckig-konfluierender* Konsolidierungen. Hier wird zwar die Grenze der einzelnen Lungensegmente respektiert, jedoch sind häufig mehrere Lungenlappen befallen. Das *positive Aerobronchogramm* entsteht, wenn Bronchialwege von dicht konsolidierten Lungenabschnitten ummauert sind.

**Figure 4:**
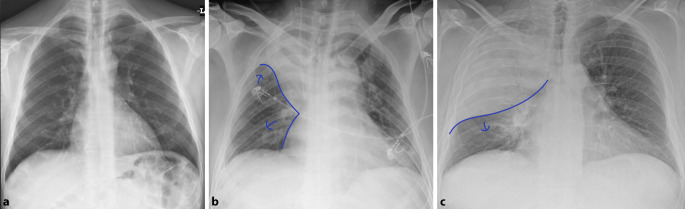

Differenzialdiagnostisch sollte man bei solchen homogenen Verschattungen die segmental oder lobär begrenzt sind, eine *Atelektase* in Betracht ziehen. Hinweise auf einen Volumenverlust, wie ein Mediastinalshift oder Verziehungen der interlobären Septen, respektive des Diaphragmas sind für die Unterscheidung hilfreich. Dem gegenüber steht die Volumenzunahme durch ein pneumonisches Infiltrat. Auch hier kann es zu Verschiebungen, in diesem Fall Ausbuchtungen, der Interlobärsepten kommen, dem „bulging fissure sign“ (Abb. 4). Pathognomonisch ist es für *Klebsiella-pneumoniae*-Infektionen, hier vor allem im rechten Oberlappen lokalisiert. Die Prävalenz dieses Zeichens ist jedoch insgesamt rückläufig. Nichtinfektiöse Ursachen für eine „bulging fissure“ können alveoläre Hämorrhagien, Tumoren und Abszesse sein.

**Figure 5:**
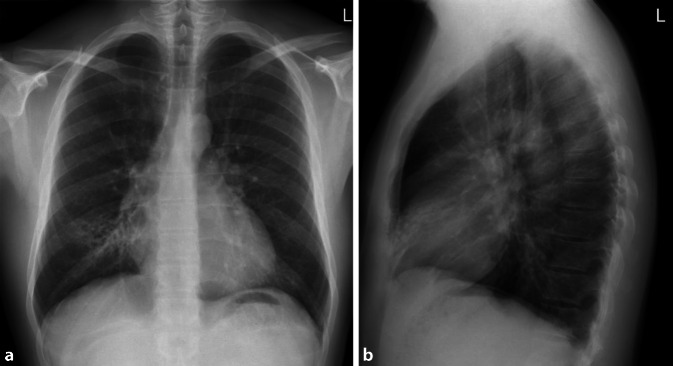

Das *Silhouettenzeichen* wurde erstmals beschrieben, um Pathologien jeglicher Art anatomisch zuzuordnen. Es beschreibt den Verlust der normalen Grenzflächen zwischen der verdichteten Lunge und Weichteilen und kann durch jeglichen Pathomechanismus ausgelöst werden, bei dem Luft in den Bronchien ersetzt wird [13]. Der Verlust der Grenzflächen, tritt im Röntgenbild immer dann auf, wenn sich die beiden Strukturen gleicher Dichte auf derselben anatomischen Ebene befinden. Ein entzündliches Infiltrat im Mittellappen weist ein Silhouettenphänomen gegenüber dem rechten Herzschatten auf und kann so von einer Konsolidation im rechten Unterlappen differenziert werden. Gleiches gilt für die Lingula und den linken Herzrand (Abb. 5). Nichtinfektiöse Ursachen für ein positives Silhouettenzeichen können Atelektasen, Aspiration, Pleuraergüsse oder Tumoren sein.

**Figure 6:**
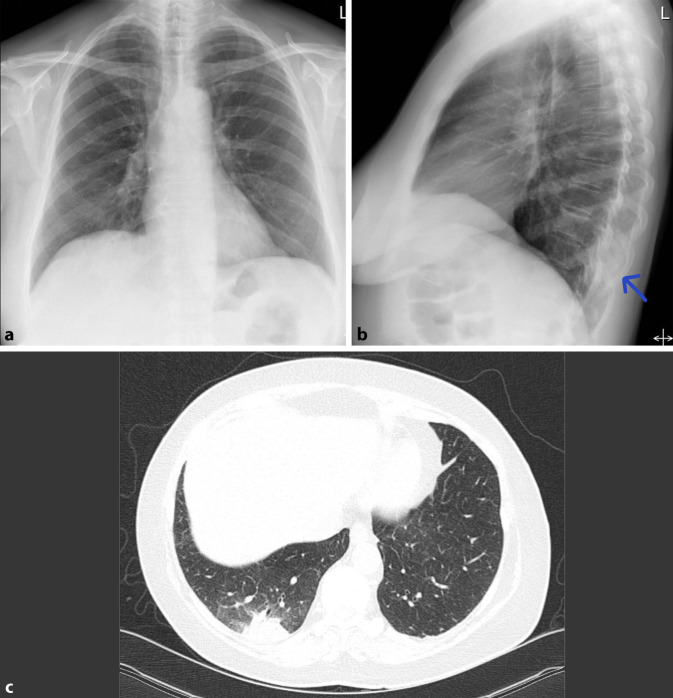

Ein weiteres häufig beschriebenes, jedoch unspezifisches Zeichen ist das „spine sign“ im lateralen Strahlengang. In einer normalen Röntgen-Thorax-Untersuchung nimmt die Transparenz der Wirbelkörper zum Zwerchfell hin zu. Eine Transparenzerhöhung spricht bei geringer Sensitivität mit hoher Spezifität für eine Pathologie im Unterlappen ([14]; Abb. 6).

**Figure 7:**
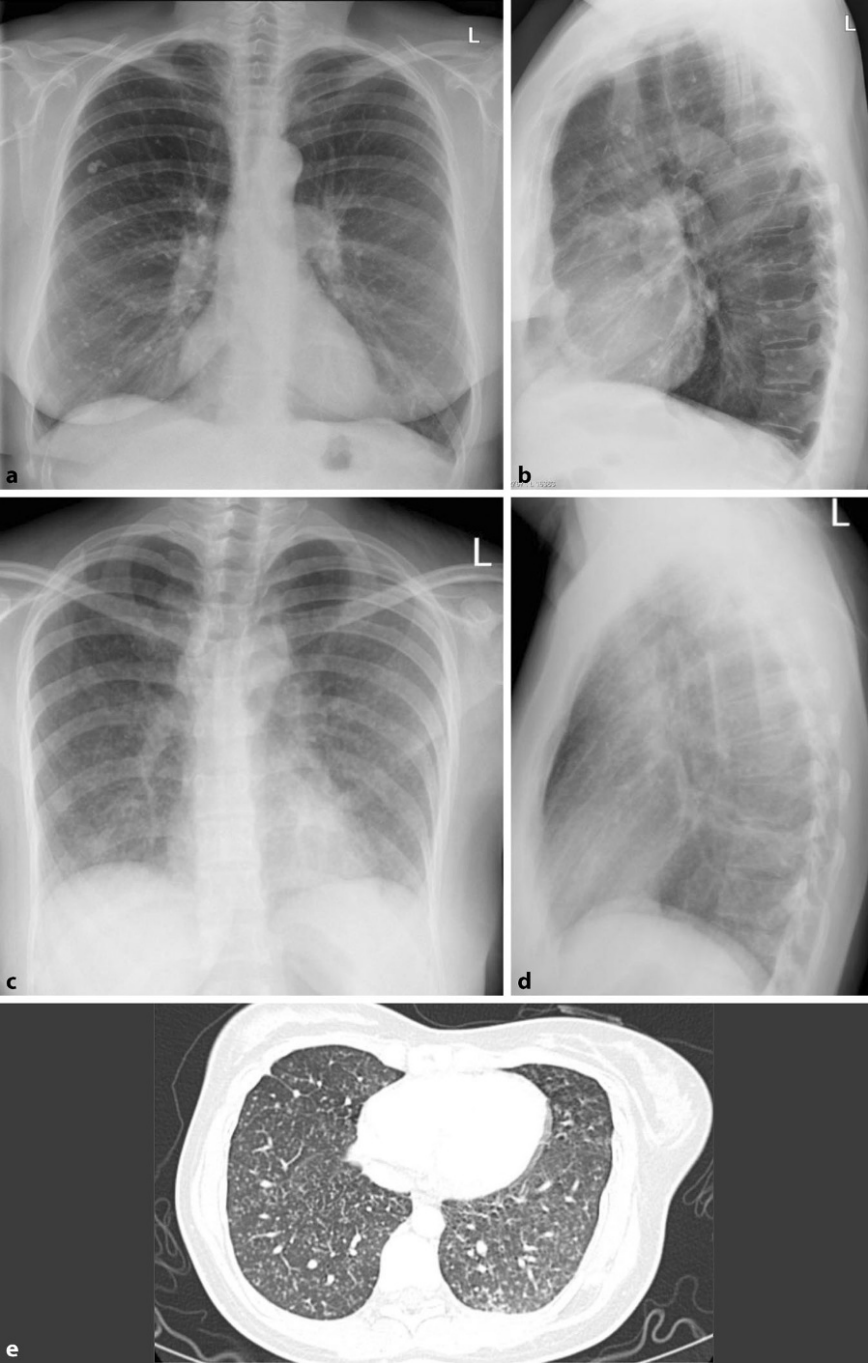

Als Sonderform des alveolären Musters, ist die *miliare Verteilung* zu nennen. Sie ist gekennzeichnet durch multiple kleinste pulmonale Noduli (< 3 mm). Eine miliare (Milium, *lat.:* „Hirsekorn“) Verteilung spricht für eine hämorrhagische Streuung des Erregers und ist pathognomonisch für die Tuberkulose, kann jedoch bei immunsupprimierten Patienten auch durch andere Erreger ausgelöst werden; exemplarisch zu nennen ist hier die Histoplasmose oder Varizellen-Pneumonie (Abb. 7). Differenzialdiagnostisch ist an eine diffuse Metastasierung primär bei Melanomen, Schilddrüsen- oder Nierenzellkarzinomen zu denken. Chronische Verschattungen miliaren Musters können selten Zeichen einer Sarkoidose oder einer Pneumokoniose sein und sollten bei entsprechenden Risikofaktoren ebenfalls in Betracht gezogen werden [15].

**Figure 8:**
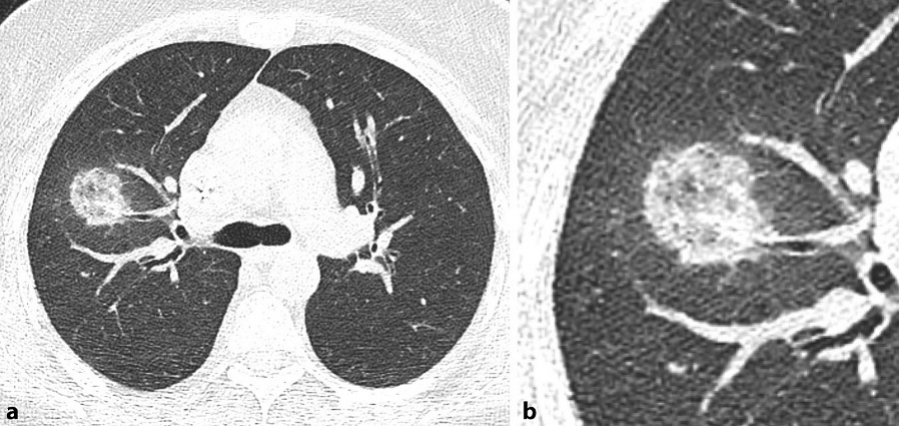

Pilze grenzen sich durch ein eigenes Erscheinungsbild ab. Sie stellen sich als, teils große, pulmonale Rundherde dar. In der Computertomographie sieht man angrenzend an diesen Rundherd häufig milchglasartige Veränderungen, welche an einen Heiligenschein erinnern; das „halo sign“ ist pathognomonisch für eine Aspergillus-Infektion ([10]; Abb. 8).

Während o. g. Muster (Tab. 2) häufig unspezifisch sind, kann ein begleitender Pleuraerguss meistens ausreichend und mit hoher Verlässlichkeit beurteilt werden. Charakteristisch ist die homogene Verschattung des kostodiaphragmalen Recessus im lateralen Strahlengang [16].

**Table 2:** 

Zeichen	Diagnose	Differenzialdiagnose
Konsolidierungen (± Aerobronchogramm)	Pneumonie	Atelektase, Tumor, Aspiration
Silhouettenzeichen	Pneumonie	Atelektase, Tumor, Erguss
„Bulging fissure sign“	Lobärpneumonie	Abszess, Tumor, Blutung
Ground-glass-Opazitäten	Atypische Pneumonie	Lungenödem, Tumor, Vaskulitis
„Halo sign“	Aspergillus	Pseudomonas, HSV, CMV, Granulomatose mit Polyangiitis
Luft-Flüssigkeits-Spiegel	Empyem, Abszess	Tumor(nekrose), Granulomatose mit Polyangiitis
„Spine sign“	Pneumonie	Atelektase, Erguss, Tumor
Miliares Muster	Tuberkulose	Varizellen, Metastasen

*HSV* Herpes-simplex-Virus, *CMV* Zytomegalie-Virus

**Figure 9:**
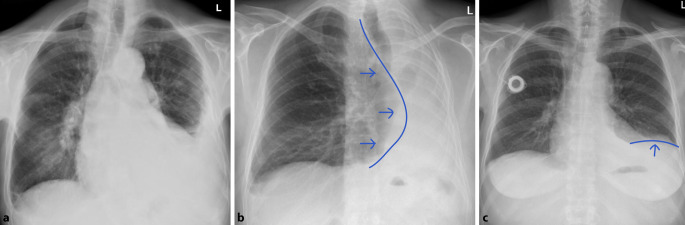

Differenzialdiagnostisch muss bei größeren Verschattungen an einen Hämatothorax oder eine (Teil‑)Pneumektomie gedacht werden, was sich mit einem Blick in die Patientenakte schnell evaluieren lässt (Abb. 9).

Schwieriger ist es bei der Differenzialdiagnose *Pleuraempyem*. Das typische Muster ist eine linsenförmige Verdickung der Pleura mit Aussparung des Recessus. Jedoch ist eine Beurteilung auch mit einer ergänzenden CT-Untersuchung oft nicht konklusiv möglich. Zusätzliche Zeichen wie eine pleurale Verdickung oder eine vermehrte Kontrastmittelanreicherung treten oft erst spät auf [17].

**Figure 10:**
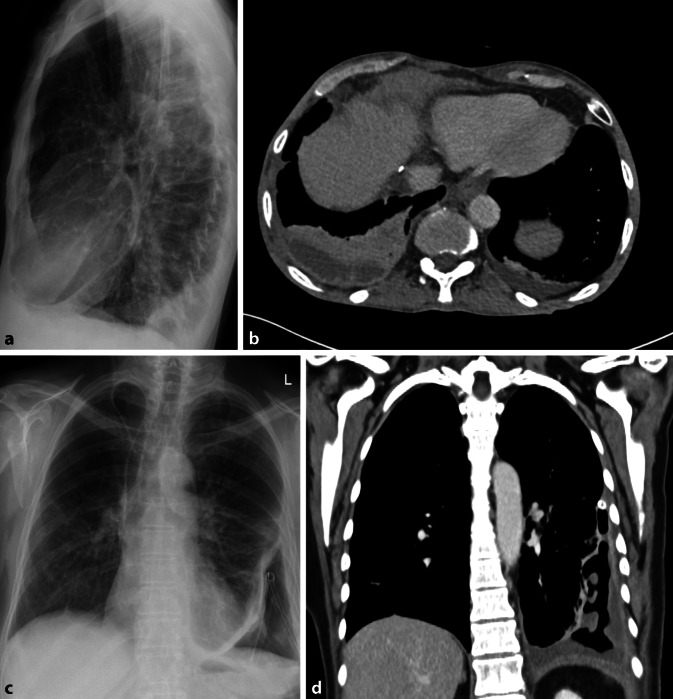

Komplikationen des Empyems, respektive der fulminanten Pneumonie, ist die bronchopleurale Fistel. Bereits im Röntgenbild würde sich dies durch einen Luft-Flüssigkeits-Spiegel im pleuralen Raum darstellen. Differenzialdiagnostisch ist bei Nachweis eines Luft-Flüssigkeits-Spiegels jedoch auch an einen Lungenabszess mit gasbildenden Bakterien zu denken ([15]; Abb. 10).

## Rolle der Computertomographie – wann ist sie notwendig?

**Figure 11:**
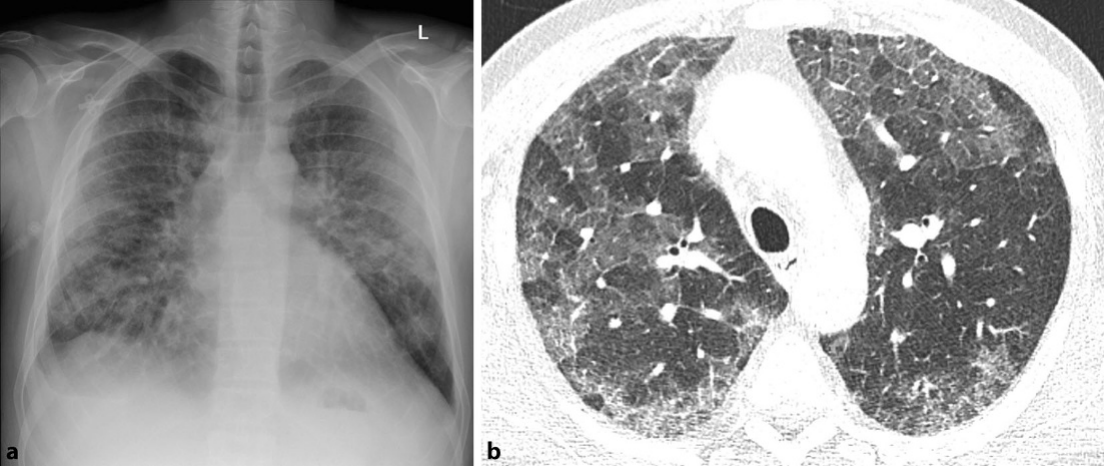

Fehlende Veränderungen in der Thoraxübersichtsaufnahme allein stellen keine Indikation für eine ergänzende CT-Untersuchung dar. Im klinischen Alltag erschweren jedoch zunehmend resistente und atypische Erreger, insbesondere im nosokomialen Umfeld, Therapie und Diagnostik. Tumorpatienten, chronisch Kranke oder Patienten unter Immunsuppression sind anfällig für Erreger, welche für den immunkompetenten Patienten in den meisten Fällen harmlos sind. Als häufigster opportunistischer Erreger ist hier Pneumocystis jirovecii zu nennen. Während bei der Pneumocystis-jirovecii-Pneumonie (PJP) die Röntgenaufnahme des Thorax häufig unauffällig ist, weist die CT-Untersuchung charakteristisch Bild eines „crazy paving“ auf. Das typische Mischbild aus bilateralen Ground-glass-Opazitäten und Konsolidierungen, meist zentral oder im Oberlappen betont, wird oft begleitet von irregulär verdickten Septen, Kavitäten und Zysten und erinnert mit seinem Muster an ein Mosaik (Abb. 11; [18]).

**Figure 12:**
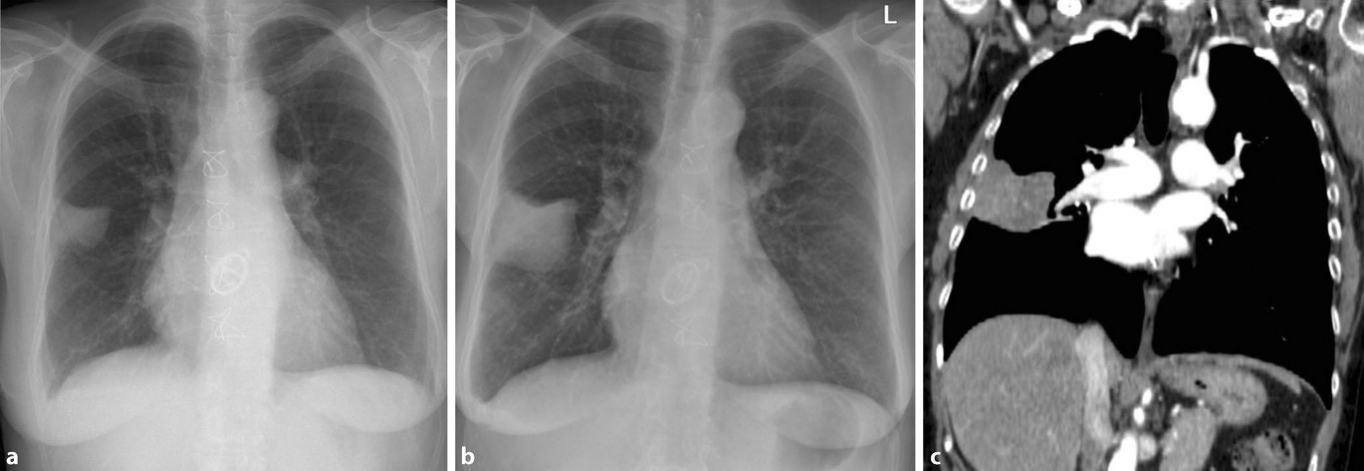

Beim immunkompetenten Patienten spielt die Computertomographie bei persistierender Symptomatik insbesondere in der Reevaluation der Therapie eine wichtige Rolle [19]. Bei chronischen und progredienten Konsolidierungen oder „red flags“ wie Hämoptoe muss differenzialdiagnostisch auch immer an ein zugrundeliegendes Bronchialkarzinom gedacht werden (Abb. 12).

## Pulmonale Rundherde – nur eine Entzündung?

Rundliche Transparenzminderungen mit einer Größe bis 3 cm werden als pulmonale Rundherde bezeichnet. Größere Läsionen, gelten bereits als Raumforderung und somit primär als weiter abklärungsbedürftig. Eine dezidierte Abklärung mittels Computertomographie ist hier notwendig [20].

Die Prävalenz von malignen Läsionen variiert je nach Quelle sehr stark. Nur bei 2–20 % der singulären Rundherde handelt es sich um die Primärmanifestation eines Bronchialkarzinoms. Mehr als die Hälfte der inzidentellen Läsionen sind benigne, wie z. B. Hamartome, Tuberkulome oder Granulome, vaskuläre Läsionen wie Infarkte und Hämatome oder Entzündungen [21]. Morphologische Charakteristika können bei der Risikoeinschätzung pulmonaler Rundherde helfen. Verkalkungsmuster in benignen Läsionen sind jedoch oft unspezifisch, nicht vorhanden oder falsch-positiv. So können einige Bronchialkarzinome ebenfalls atypische Verkalkungen aufweisen, welche als Benignitätskriterium fehlinterpretiert werden können [21].

## Limitationen des konventionellen Bildes

**Figure 13:**
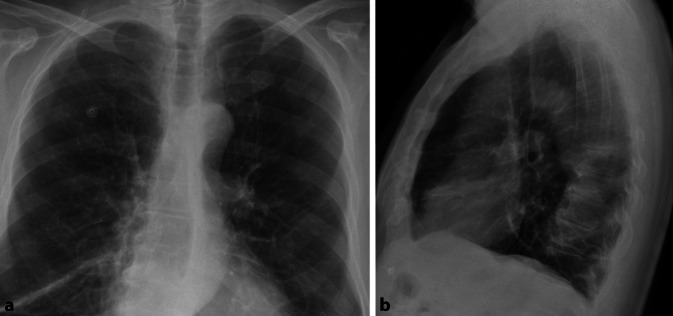

Obwohl das konventionelle Röntgen-Thorax-Bild immer noch zu den Standardverfahren in der Primärdiagnostik gehört, ist seine Sensitivität in der Detektion von pulmonalen Rundherden deutlich geringer als die der Computertomographie, daher wird auch die Niedrigdosis-CT und nicht die konventionelle Röntgen-Thorax-Untersuchung in Lungenkarzinomfrüherkennungsprogrammen verwendet [22]. Bei falscher Durchführung ist die Röntgen-Thorax-Bildgebung aufgrund ihrer Zweidimensionalität deutlich eingeschränkt [5]. Pseudoläsionen können extrapulmonale oder kutane Läsionen, Devices oder Frakturen sein, welche sich als Lungenrundherd darstellen; sie machen 20 % der Lungenopazitäten aus [23]. Standardvorgehen ist deshalb immer die Aufnahme in zwei Ebenen. So lässt sich durch die ergänzende seitliche Thoraxaufnahme schnell zwischen einem Rundherd in der Mamma oder einer extrathorakalen Installation und einem pulmonalen Nodulus unterscheiden (Abb. 13). Ist eine Läsion lediglich in einer Ebene darstellbar, sollte man von dem Vorliegen einer Pseudoläsion ausgehen. Ein weiteres Kriterium zur Differenzierung von Pseudoläsionen und pulmonaler Rundherde ist die Berandung. Während Pseudoläsionen keine vollständige Berandung zeigen, sind pulmonale Rundherde vollständig von Lungenparenchym umgeben.

**Figure 14:**
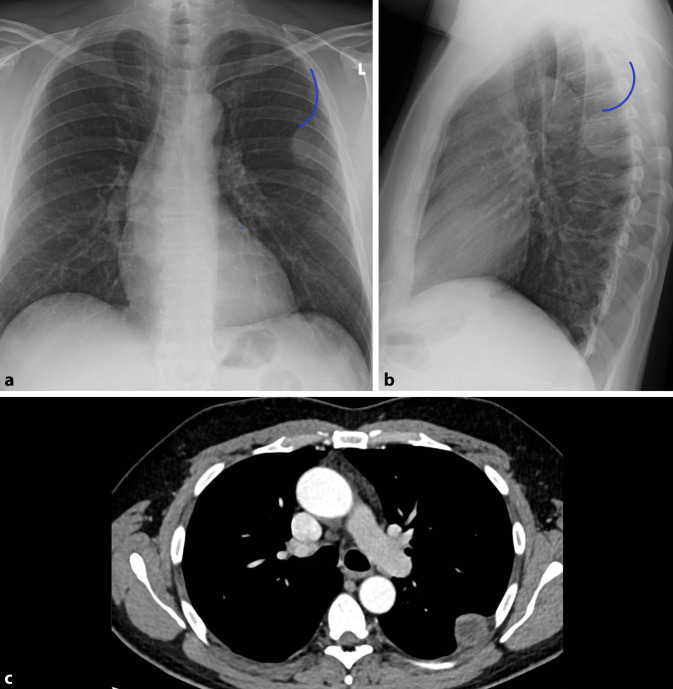

Ob eine Läsion innerhalb des Lungenparenchyms oder doch extrathorakal liegt, lässt sich an dem gemessen Winkel zur Pleura leicht erkennen. Während intrapulmonale Läsionen einen spitzen Winkel zeigen, ist er bei Pseudoläsionen flach (Abb. 14).

**Figure 15:**
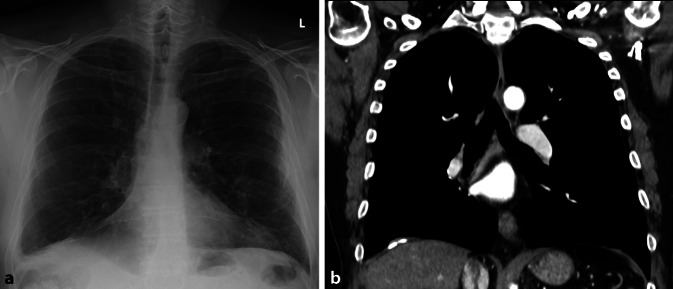

Pleurale Läsionen zählen per Definition ebenfalls zu Pseudoläsionen. Häufig handelt es sich dabei um benigne Plaques. Prädilektionsstellen sind insbesondere die posterolateralen, mediastinalen und diaphragmalen Pleuraabschnitte, die Lungenapeces und kostodiaphragmatischen Winkel bleiben meistens ausgespart. Charakteristische Verkalkungen erleichtern die Diagnose, fehlen jedoch in bis zu 95 % der Pleuraplaques (Abb. 15). Risikofaktor für die Entstehung pleuraler Plaques ist die Berufsexposition mit Asbest, eine maligne Entartung mit einem meist fulminanten Krankheitsverlauf ist möglich [24, 25].

## Hounsfield-Units: Rolle der Computertomographie

Durch die Einführung der Computertomographie kam auch die Möglichkeit der Dichtemessung, angegeben in Hounsfield-Units (HU). Diese liefert uns Informationen nicht nur über die Morphologie, sondern auch über die Beschaffenheit einer Läsion. Fett, Flüssigkeit und Verkalkungen zeigen charakteristische HU-Werte und können bei der Klassifizierung von pulmonalen Rundherden hilfreich sein [21]. Einen weiteren Vorteil, den die CT aufbringt, ist die gleichzeitige Beurteilung der umliegenden Strukturen. Wo ein Röntgenbild wenig Auskunft bietet, kann in der CT eine genauere Beurteilung von Weichteilen, Lungenparenchym und ossären Strukturen zum Staging erfolgen. Auch die genaue Lagebeziehung zu angrenzenden Bronchien für die Evaluation einer transbronchialen Biopsie oder CT-gesteuerten Punktion kann beurteilt werden. In fortgeschrittenen Stadien ist vor allem die Infiltration angrenzender Strukturen von prognostischer Bedeutung. Durch die in den letzten Jahren immer besser werdende Technologie wird auch mit einer Low-dose-CT bei deutlicher Dosiseinsparung (Thorax-CT 5,5 mSv vs. Low-dose-CT < 1 mSv vs. konventionelle Röntgen-Thorax-Aufnahme mit 0,10 mSv; [1]) eine hohe diagnostische Sensitivität erreicht [26]. Dies ermöglicht engmaschige Verlaufskontrollen suspekter Rundherde in der CT und erleichtert dem Radiologen die Beurteilung.

Ein individuelles Assessment bei entsprechendem Risikoprofil zur Evaluation des weiteren Vorgehens ist wichtig und wurde in den letzten Jahren nach mehreren Kriterien optimiert. Die am häufigsten verwendeten Kriterien zur Risikoevaluation von pulmonalen Runderden sind die Fleischner-Kriterien. Berücksichtigt werden hier die Anzahl und das Verteilungsmuster, die Morphologie (Größe, Form und Dichte), aber auch Risikofaktoren, welche ein Bronchialkarzinom begünstigen. Ein häufiger Fehler insbesondere bei jungen Kollegen ist die falsche Messung eines Nodulus (Längsachse × Kurzachse /2, wobei beide Messungen orthogonal zueinander erfolgen), welche bei entsprechender Größendynamik Behandlungskonsequenzen nach sich ziehen kann. Tab. 3 gibt einen Überblick über die empfohlenen Verlaufsintervalle bei soliden pulmonalen Noduli gemäß Fleischner Society. Bei immunsupprimierten Patienten jedoch sollten die Kriterien nicht angewendet werden, obwohl diese ein erhöhtes Risiko für ein Tumorleiden aufweisen [27].

**Table 3:** 

	Low-risk	High-risk^a^
< 6 mm	Kein Follow-up	Optionale CT in 12 Monaten
6–8 mm	Solitär: CT in 6–12 und optional in 18–24 Monaten Multipel^b^: CT in 3–6 Monaten, optional in 18–24 Monaten	Solitär: CT in 6–12 Monaten und 18–24 Monaten Multiple: CT in 3–6 Monaten und 18–24 Monaten
> 8 mm	Solitär: Intervall CT in 3 Monaten *oder* PET/CT *oder* Biopsie Multiple: CT in 3–6 Monaten, optional 18–24 Monaten	Solitär: Intervall CT in 3 Monaten *oder* PET/CT *oder* Biopsie Multiple: CT in 3–6 Monaten und 18–24 Monaten

*CT* Computertomographie, *PET* Positronen-Emissions-Tomographie

^a^ Risikofaktoren: Nikotinabusus, Exposition zu Asbest, Radon oder Uran, Lungenkrebs in der Familienanamnese, hohes Alter, weibliches Geschlecht, Ethnie (schwarzafrikanische oder hawaiianische Bevölkerung), spikulierte Berandung, Lokalisation im Oberlappen, Anzahl < 5, Lungenemphysem oder -Fibrose

^b^ Bei multiplen Noduli zählt der größte Nodulus

## Die Radiologie im Wandel – Zukunftsaussichten

Neue Möglichkeiten bietet die in den letzten Jahren immer häufiger angewendete Hybridbildgebung. Hier kann nicht nur die Morphologie eines Rundherdes beurteilt werden, sondern viel wichtiger auch seine biologischen Eigenschaften. So kann es in der CT allein oft schwierig sein, zwischen einer Entzündung, einem Malignom oder postoperativen, respektive posttherapeutischen Veränderungen zu unterscheiden. In Deutschland gehört die Positronen-Emissions-Tomographie(PET)/CT bereits zur Standarddiagnostik von Bronchialkarzinomen und ist zum Tumorstaging kassenärztlich anerkannt. Insbesondere in der Detektion von Fernmetastasen zeigt sie eine deutlich höhere Sensitivität [28]. In den letzten Jahren hat die künstliche Intelligenz (KI) immer mehr Einzug in die Medizin gefunden. Sogenannte CAD(„computer-aided detection“)-Systeme gehören in vielen Kliniken bereits zum Untersuchungsstandard. Neueste Studien haben gezeigt, dass neuronale Netzwerke von Computern mit hoher diagnostischer Genauigkeit pulmonale Noduli erkennen und diese hinsichtlich ihrer Malignitätswahrscheinlichkeit einordnen können [29]. Zum aktuellen Zeitpunkt dienen diese Systeme jedoch eher der Qualitätskontrolle des radiologischen Befundes und sind noch nicht in den klinischen Alltag integriert.

## Fazit für die Praxis

- Das Röntgenbild ist durch seine schnelle Verfügbarkeit weiterhin der Goldstandard in der Primärdiagnostik von Pneumonien.
- Fortschrittliche Techniken erlauben Computertomographie(CT)-Untersuchungen mit vergleichbar geringer Dosis und gewinnen somit an Bedeutung.
- Verteilungsmuster können Hinweise auf die Pathogenese einer Pneumonie liefern, sind häufig jedoch unspezifisch.
- Die Pneumonie ist eine klinische Diagnose. Radiologische Zeichen können im frühen Stadium fehlen.
- Pseudoläsionen sind häufig, können jedoch einfach von pulmonalen Rundherden unterschieden werden.
